# Com1847618plete mitochondrial genome of *Pnyxia scabiei* (Diptera: Sciaridae)

**DOI:** 10.1080/23802359.2020.1847618

**Published:** 2021-01-19

**Authors:** Caixia Liu, Qingyun Wang, Hong Wu, Junhao Huang

**Affiliations:** Department of Forestry Protection, School of Forestry and Biotechnology, Zhejiang A&F University, Hangzhou, Zhejiang, China

**Keywords:** Potato scab-gnat, phylogenetic analysis, Holarctic species, Sciarioidea

## Abstract

The potato scab-gnat, *Pnyxia scabiei*, was recorded as a pest attacking potato tubers and greenhouse cucumber plants. The mitochondrial genome of a total length of 15,437 bp was sequenced, including 13 protein-coding genes, 22 tRNA genes, and two rRNA genes with A + T content of 77.2%. Six gene overlaps were found from 1 to 34 bp. Phylogenetic analysis showed that *P. scabiei* was closely related to *Trichosia lengersdorfi* + *Sciara ruficauda*. The study provided further data for species diversification in Sciaridae.

Sciaridae is one of the most species diverse families in Diptera, with more than 2,800 species recorded worldwide (Yang et al. 2019). Sciarids mostly live in forests and other moist shady area. The larvae feed on mycelium, saprophagous wood, rotting vegetable matter and organic matter in soil (Miao et al. 2020). *Pnyxia scabiei* is a Holarctic species, found to occur on potato tubers, greenhouse cucumber plants, and edible mushrooms, mainly damaging greenhouse crops (Gui 1933; Broadley et al. 2018). Previous studies on this species mostly concentrated on taxonomic descriptions and biological habits. However, female wings of *P. scabiei* were reduced, and male wings with m–cu crossvein present and vein M + CuA absent. Due to these exceptional morphological characters, the phylogenetic status of the genus remains controversial. Mitochondrial DNA is considered a valuable molecular marker and widely used in insect systematic studies.

Specimens were collected on the rotten root of *Platycodon grandifloras* in Songxian County, He’nan Province of China (33°53′7″N, 112°10′6″E) in April 2020. Voucher specimens of male and female adults were deposited in Zhejiang A&F University, China (Sample ID: SMLCX001-1–12; BIN. BOLD:AEA9290 and AEA9289). The genomic DNA was extracted from the whole bodies of six females using the DNeasy Blood & Tissue kit (Qiagen Hilden, Germany). Mitogenome was initially annotated using the MITOS (Bernt et al. 2013). The base composition and codon usages were analyzed using MEGA 7.0 (Kumar et al. 2016). A phylogenetic tree was performed using PhyloSuite (Zhang et al. 2020).

The complete mitogenome of *P. scabiei* (GenBank accession no. MT991051) is 15,437 bp in length, with an A + T content of 77.2%. The mitogenome is circular and contains 37 genes, 13 PCGs, 22 transfer RNAs (tRNAs), and two ribosomal RNAs (rRNAs). All genes of *P. scabiei* show the same locations and strands as *Sciara ruficauda* from the same family. Six gene overlaps were found from 1 to 34 bp, with the longest overlap between *trnL1* and *rrnL*. The total length of all 13 PCGs is 11,191 bp. The start codons of PCGs are ATT (*cox2*, *atp8*, *nad3*, *nad4l*), ATA (*nad1*, *nad2*, *nad5*, *nad6*), ATG (*nad4*, *atp6*, *cob*), AAT (*cox1*), and GTG (*cox3*). The stop codons of PCGs are TAA (*nad2*, *cox1*, *cox2*, *atp8*, *atp6*, *cox3*, *nad3*, *nad4*, *nad4l*, *nad6*, *cob*), TAG (*nad1*), and TTA (*nad5*). The 22 tRNAs size varies from 63 to 70 bp, while 12S and 16S rRNAs are 791 and 1,371 bp in length, respectively.

The phylogenetic tree was performed using maximum likelihood (ML) and Bayesian inference (BI), based on all PCGs of eight mitogenomes (Figure 1). The results revealed that *P. scabiei* was closely related to Sciarinae (*Trichosia lengersdorfi* + *Sciara ruficauda*), with a great genetic distance. The genus was placed in the subfamily Cratyninae by Menzel and Mohrig (2000) based on morphological characters, although it differs in wing veins and posterior pronotum setae. However, the placement was not yet supported by molecular data, since the mitogenome of the type genus (*Cratyna*) of the subfamily is not available. The mitogenome provides fundamental information for studying molecular phylogeny and evolution.

**Figure 1.**
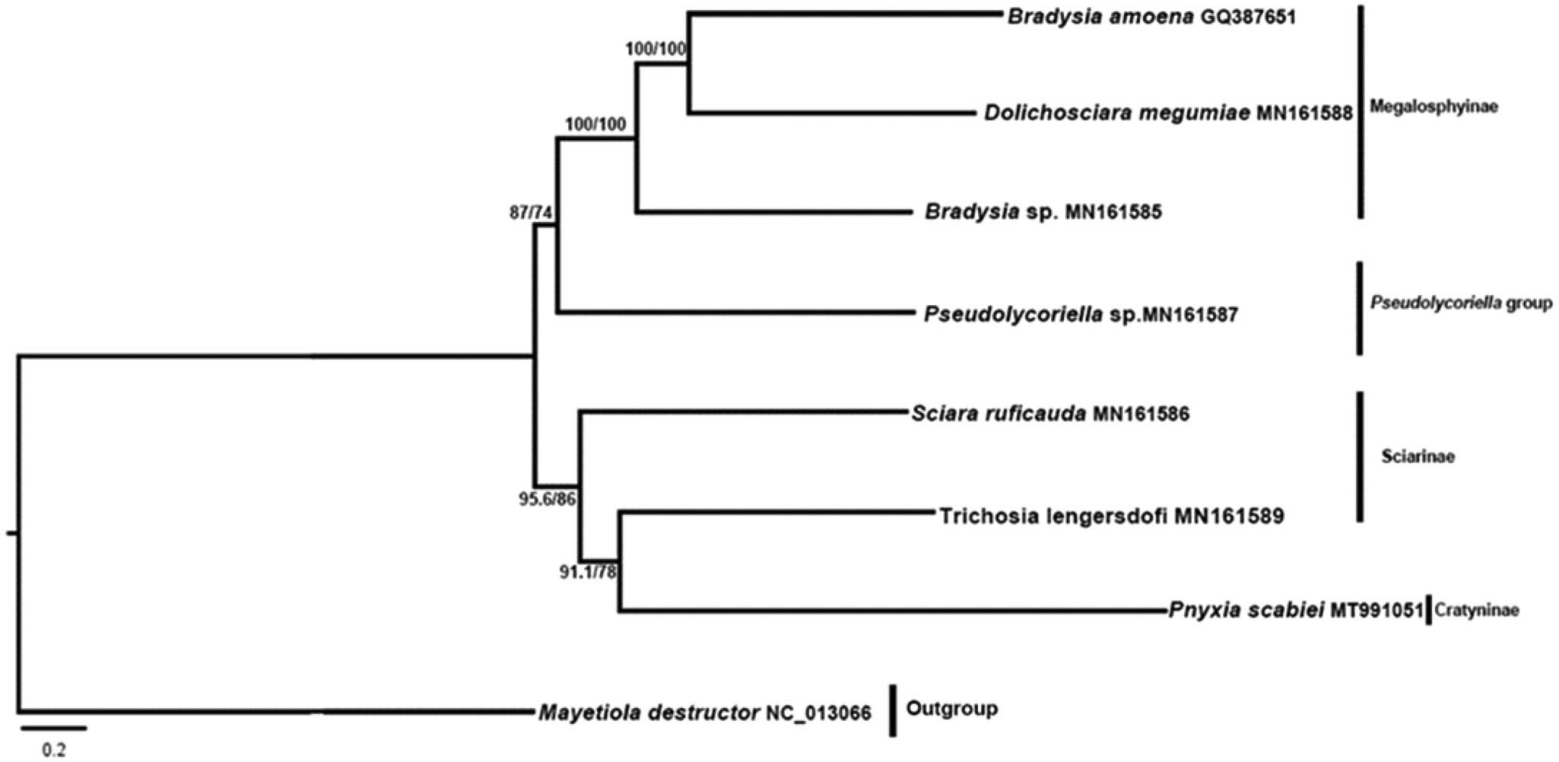
Phylogenetic tree of seven species of Sciaridae. The numbers on branches refer to the posterior probabilities (PP) and SH-aLRT values (%). Clades are labeled with subfamilies or groups of the family.

## Data Availability

The mitogenome sequences of this study is openly available in GenBank (accession number MT991051, https://www.ncbi.nlm.nih.gov/nuccore/MT991051.1/).
